# Retinoblastoma: Etiology, Modeling, and Treatment

**DOI:** 10.3390/cancers12082304

**Published:** 2020-08-16

**Authors:** Rossukon Kaewkhaw, Duangnate Rojanaporn

**Affiliations:** 1 Section for Translational Medicine, Faculty of Medicine Ramathibodi Hospital, Mahidol University, Bangkok 10400, Thailand; 2 Department of Ophthalmology, Faculty of Medicine Ramathibodi Hospital, Mahidol University, Bangkok 10400, Thailand; Duangnate.roj@mahidol.ac.th

**Keywords:** pediatric cancer, retinoblastoma, genomics, transcriptomics, cancer cell-of-origin, disease model, clinicopathology, globe preservation, targeted therapy

## Abstract

Retinoblastoma is a retinal cancer that is initiated in response to biallelic loss of *RB1* in almost all cases, together with other genetic/epigenetic changes culminating in the development of cancer. *RB1* deficiency makes the retinoblastoma cell-of-origin extremely susceptible to cancerous transformation, and the tumor cell-of-origin appears to depend on the developmental stage and species. These are important to establish reliable preclinical models to study the disease and develop therapies. Although retinoblastoma is the most curable pediatric cancer with a high survival rate, advanced tumors limit globe salvage and are often associated with high-risk histopathological features predictive of dissemination. The advent of chemotherapy has improved treatment outcomes, which is effective for globe preservation with new routes of targeted drug delivery. However, molecularly targeted therapeutics with more effectiveness and less toxicity are needed. Here, we review the current knowledge concerning retinoblastoma genesis with particular attention to the genomic and transcriptomic landscapes with correlations to clinicopathological characteristics, as well as the retinoblastoma cell-of-origin and current disease models. We further discuss current treatments, clinicopathological correlations, which assist in guiding treatment and may facilitate globe preservation, and finally we discuss targeted therapeutics for future treatments.

## 1. Introduction

Retinoblastoma is a tumor that develops in the retina, which is diagnosed in the first few years of a child’s life, affecting approximately 1 in 16,000 live births [1,2]. The tumor is initiated through biallelic loss of tumor suppressor gene *RB1* in more than 95% of cases [1], and develops after additional genetic/epigenetic changes [3,4,5]. Once the tumor develops, a white pupillary reflex known as leukocoria is the first readily observed sign and is commonly detected among patients [6]. Non-hereditary retinoblastoma comprises the majority of cases (60%) with both *RB1* alleles locally mutated in the affected retina [2,7]. Hereditary retinoblastoma (40%) is associated with a *RB1* germline-predisposing variant, and subsequent somatic inactivation of the other allele [2,7]. For this reason, cases of non-heritable retinoblastoma have unilateral tumors, unlike heritable retinoblastoma that often develops bilaterally and multifocally.

Retinoblastoma is a curable cancer with ocular survival when treated promptly, but it is universally fatal if left untreated. Tumors become advanced if treatment is delayed, hindering vision and globe salvages with a risk of metastasis [8,9,10]. Chemotherapy is the standard of care for patients with a high risk of metastasis but has inevitable toxicity [10,11,12,13,14,15]. Such treatment is also commonly used for globe sparing with different routes of drug delivery depending on the clinical features and anticipated outcomes [16]. Similar to other cancers, therapeutics that selectively target tumors, while sparing the normal retina, are desired with the expectation of improving treatment outcomes.

Which cell type is the origin of retinoblastoma, why such cells are so vulnerable to malignant transformation when *RB1* is lost, and how the disease progresses are the outstanding questions that must be answered to ultimately benefit designing targeted therapies. Evidence suggests that retinoblastoma genesis is unique in humans [17]. Thus, disease models should be appropriately used to study the disease and test therapies [17]. Moreover, advanced imaging to detect and monitor tumoral events, clinicopathological studies, and the advent of chemotherapy with different routes of drug delivery have facilitated and improved tumor control and globe retention [16,18]. This review provides the current knowledge on the etiology, modeling, and treatment of retinoblastoma with particular attention to (i) the molecular and cellular basis concerning genomic and transcriptomic landscapes with correlations to clinicopathological characteristics, and the tumor cell-of-origin; (ii) disease models with the features and limitations of each model; and (iii) current treatments and clinicopathological correlations regarding histopathological risk features, which assist in guiding treatment and may facilitate globe preservation, and assessment of targeted therapeutics for future treatments.

## 2. Molecular and Cellular Basis of Retinoblastoma

### 2.1. Genomic Landscape

The cellular consequences of *RB1* inactivation have been associated with multiple forms of genomic instability, which fuels tumorigenesis by eliminating safeguards that limit oncogenic transformation [19,20,21,22]. Such genomic instability is a hallmark of almost all types of cancers. However, compared with other cancers including *RB1*-deficient malignancies, retinoblastoma exhibits minimal genomic instability because of unique cell type-specific circuitry [5,23,24,25,26]. Genomic studies to define drivers other than *RB1* that promote progression have been primarily conducted by profiling large genomic changes using array techniques (Table 1). Genomic alterations in retinoblastoma are unique with common gains at 1q, 2p, and 6p and loss at 16q. The 6p (44–69%) or 1q (38–70%) gains are most frequently identified in tumors, followed by the 2p gain (15–43%) and 16q loss (18–46%) [4,27,28,29,30,31,32]. The candidate drivers are subsequently defined by minimally overlapping regions of each common gain or loss and include *MDM4*, *KIF14* in 1q, *MYCN* in 2p, *DEK*, *E2F3*, *ID4* and *SOX4* in 6p, and *CHD11* and *RBL2* in 16q (Table 1). Interestingly, the 1q gain and 16q loss in tumors are indicative of increased levels of genomic instability and associated with late diagnosis and non-hereditary retinoblastoma [27,28,31,33]. Recurrent loss of 16q is believed to impair candidate suppressor genes and is implicated in advanced disease [34,35,36]. *MYCN* gain/amplification occurs in approximately 8% of retinoblastoma and is included in the most common focal genomic aberration [24,37,38]. A copy number of *MYCN* greater than 28 is considered high amplification and is associated with *RB1*+/+ or *RB1*+/− retinoblastoma [37,39]. High *MYCN* amplification is proposed as a novel mechanism of disease initiation in retinoblastoma without *RB1* mutation [39].

Studies have examined small genetic lesions by exon sequencing and revealed that mutations beyond *RB1* are rare in retinoblastoma. Only *BCOR* and *CREBBP* were recurrently mutated at 10–13% and 4% of total tumors, respectively [5,23]. Similarly, these genes were identified to have focal loss in chromosomes X and 16, respectively [24]. Nevertheless, a recent study reported recurrent mutations in *BCOR*, *ARID1A*, *MGA*, *FAT1*, and *ATRX* and association of these mutations with aggressive histopathological features [40]. *MDM2* has been proposed as a genetic modifier in retinoblastoma [41]. Unlike its homolog *MDM4*, *MDM2* is rarely altered [42]. A single nucleotide polymorphism identified in the *MDM2* promoter may enhance *MDM2* expression that contributes to tumor susceptibility [41].

### 2.2. Gene Expression Profile of Tumor Tissue from Enucleated Eyes

Gene expression profiling can be used to determine the histogenesis of tumors and biological pathways underlying tumorigenesis, while tumor cellularity is crucial for the accurate interpretation of data [43,44,45]. Rod-associated genes are expressed abundantly in the normal human retina because of a greater proportion of rod cells compared with other retinal cells. However, tumor cells stained for rod markers are almost absent [46] in contrast to abundant numbers of cone-like cells in human retinoblastoma. Accordingly, pure tumors are defined by low expression of rod-enriched genes and high expression of cone-enriched genes compared with the normal retina [43,44,45,47,48]. Functional annotation of the gene expression profile of retinoblastoma versus the normal retina identifies genes associated with cell cycle progression, DNA replication and repair, growth and proliferation, and cell death [43,47,49].

Cone-associated genes are prominently expressed in retinoblastoma [44,45,47,48]. Kapatai et al., 2013, suggested that expression profiles stratify *RB1*-deficient retinoblastoma into two groups [44]. Group 1 exhibits expression of retinal progenitor genes and, to a lesser extent, cone genes, agreeing with McEvoy et al., 2011, showing that genes associated with the multiple retinal cell types were expressed in tumors [50]. In contrast, group 2 has enriched expression of cone genes. These data suggest that the tumor cell-of-origin is retinal progenitors in group 1 in contrast to cone cells in group 2. However, Kooi et al., 2015, proposed that, rather than clear classification, gradual changes best describe the gene expression profiles of retinoblastoma [45]. Expression of cone genes would represent a signature of retinoblastoma. However, during disease progression, tumors lose their photoreceptor signature while increasing expression of genes associated with M-phase and mRNA/ribosome synthesis [45]. Despite the different views, a common finding is that expression of cone genes is always detected in retinoblastoma [44,45]. Additionally, retinoblastoma with an enriched cone signature is associated with less genomic instability [44,45]. Genomic disruption is proposed as a mechanism underlying tumor progression, driving the loss of the cone signature while gaining expression of genes associated with retina development, which has been identified in retinal progenitors [45,50]. Depleted expression of cone genes positively correlates with severe anaplasia, a characteristic of advanced retinoblastoma [43].

### 2.3. Correlation of Genomic and Transcriptomic Profiles with Clinicopathological Characteristics

Clinicopathological features related to genomic and transcriptomic profiles of tumors may facilitate better understanding of disease progression. Correlation studies indicate that bilateral tumors are associated with an enriched cone signature, whereas unilateral tumors are associated with a reduced cone signature [33,45] (Table 2). Bilateral retinoblastoma usually develops and is diagnosed earlier than unilateral retinoblastoma [2], while the corresponding cone signature is enriched in differentiated tumors [2,33,44,45] (Table 2). This supports precedent formation of well-differentiated tumors [3,51]. Therefore, well-differentiated tumors develop first, and if diagnosed late or left untreated, may transform and become less differentiated. Less differentiated tumors that appear to lose the cone signature and genomic integrity are commonly found in older patients in contrast to younger patients carrying tumors with a high degree of differentiation [33,38,45,51,52] (Table 2). This also indicates that delayed diagnosis/enucleation significantly affects progression. However, none of the genomic and gene expression changes are associated with high-risk pathological features that are important factors to decide definitive treatments after enucleation [33,44,45] (Table 2).

Tumors with a reduced cone signature generally have a large volume, are often found in a greater eye size and located peripherally or throughout in the retina, representing advanced-stage disease [33,45,53] (Table 2). In contrast, tumors with enriched cone expression appear locally in the central retina and are found in a smaller volume and eye size [33,45,53] (Table 2). The number of tumor lesions correlates with the cone signature [53] (Table 2), which is inconsistent with a previous report [45]. Tumor volume and lesion numbers are independent of a genomic instability [33]. Interestingly, tumors with a reduced cone signature exhibit better responses to chemotherapeutic agents such as actinomycin D, doxorubicin, and carboplatin [45], concurring with chemoresistance of residual retinoma with featured photoreceptor differentiation in enucleated eyes [54,55,56] (Table 2). This implies that poorly differentiated tumors are more susceptible to death during chemotherapy.

### 2.4. Retinoblastoma Cell-of-Origin

Definitive determination of the cell-of-origin and understanding how tumor cells exploit the mechanisms of such susceptible cells for malignant transformation may facilitate development of treatments that specifically target tumor cells, while sparing normal retinal tissue. In this section, we discuss direct and circumstantial evidence supporting a cone cell-of-origin and elucidate why cone cells are susceptible to cancerous transformation.

#### 2.4.1. Cone Cells Proliferate in Response to Retinoblastoma Protein (pRb) Loss, While Cone-Enriched Genes are Prominently Expressed in Retinoblastoma

**Figure 1:**
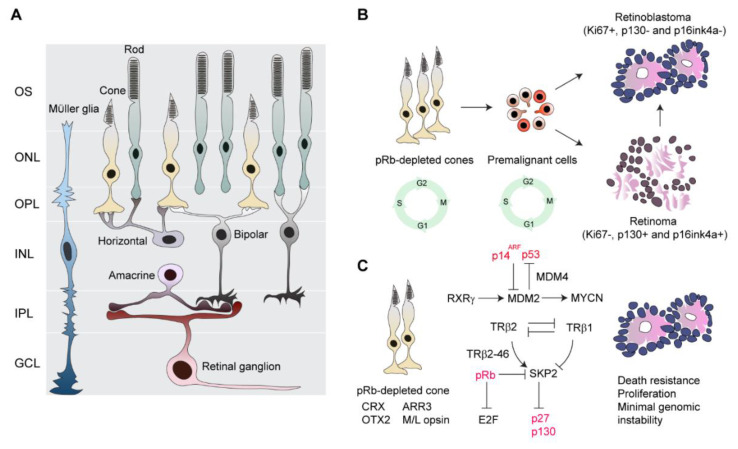
Development of retinoblastoma. (**A**) Major retinal cell types. (**B**) Retinoblastoma protein (pRB)-depleted maturing cone cells re-enter the cell cycles and become neoplastic, while premalignant cells (dark orange), which are vulnerable to cancerous transformation, proliferate highly and develop into retinoblastoma [3,17,26]. However, some other premalignant cells (weak orange) undergo cell cycle arrest and senesce, becoming retinoma that often develop into retinoblastoma [3,17]. (**C**) Cone-associated proteins are present in tumor cells [46,48], while cone circuitry facilitates development of retinoblastoma [17,25,26,46,67,68,69,70,71]. A cone-specific signal circuitry is linked with the pRB pathway through S-phase kinase associated protein 2 (SKP2), whose activity is suppressed through an N-terminal interaction with pRb [72]. SKP2 and oncoprotein Mdm2 (MDM2) are E3 ubiquitin ligase and facilitate degradation of tumor suppressor proteins p27, p130 and p53 [73,74,75]. See text for details. Red, inactive/decreased proteins; black, synthesized/active proteins. Abbreviations: OS, outer segment; ONL, outer nuclear layer; OPL, outer plexiform layer; INL, inner nuclear layer; IPL, inner nuclear layer; GCL, ganglion cell layer.

pRb is a cell cycle regulator and centrally important for tumor suppression [57]. pRb interacts physically with two critical mediators, E2F1 and SKP2, to inhibit cell cycle progression, thereby blocking G1-S phase transition [58]. In the normal retina, pRb is widely expressed in developing cells to ensure appropriate cell-cycle exit and terminal differentiation [59,60]. Differentiating cone cells, particularly in the fovea, express high levels of pRb [59]. Depletion of pRb in the developing human retina has shown that only post-mitotic cones re-enter the cell cycle and become apoptosis resistant and proliferative [17,26] (Figure 1). In contrast, progenitors and Müller glia undergo apoptotic death, while other cells, such as retinal ganglion cells, rod cells, horizontal and amacrine interneurons, and bipolar cells, remain in the post-mitotic state [26] (Figure 1A). Furthermore, pRb-depleted human cone cells that express medium/long-wave-sensitive (M/L) opsin and cone arrestin (ARR3) form retinoma and retinoblastoma-like lesions. This indicates that maturing cone cells rather than early post-mitotic, immature cone cells (ARR3-negative cells) are oncogenically activated and highlights the importance of developmental state specificity in tumorigenesis [17,26] (Figure 1B,C).

Examining retinal proteins in retinoblastoma has shown that transcription factors cone-rod homeobox protein (CRX) [61] and homeobox protein OTX2 [62], which specify fate decision for photoreceptors, are widely expressed in retinoblastoma [63]. Retinoid X receptor gamma (RXRγ) [64] and thyroid hormone receptor beta 2 (TRβ2) [65,66], which are cone fate determinants, and M/L opsin are prominently produced in most retinoblastoma cells [46,48], agreeing with immunostaining of pRb-depleted cone cells of the normal retina [17] (Figure 1). However, rod cells and other retinal cells as well as synthesis of their corresponding proteins are almost absent in retinoblastoma, while cells stained for Müller cell and astrocyte markers are consistently detected in tumors [46,48]. Cells stained positively for non-cone markers are non-neoplastic pRB-positive cells [46]. The behavior of pRB-depleted cells in the normal retina together with histogenesis of retinoblastoma favors the concept of a cone cell-of-origin (Figure 1).

#### 2.4.2. Cone-Specific Signal Circuitry Promotes Retinoblastoma Genesis

If cone cells are the cell-of-origin, why are these cells so susceptible to tumorigenic transformation? The intrinsic gene expression program of cone cells can be oncogenically activated in the absence of pRb [17,26,46]. Cone-specific signal circuitry, including TRβ2, RXRγ, oncoprotein Mdm2 (MDM2), and N-myc proto-oncogene protein (MYCN), collaboratively supports the transition from premalignant to malignant lesions [17,26,46](Figure 1C). Additionally, SKP2, an important survival signal in pRb-deficient malignancies, may be an important factor linking the pRB pathway with cone-specific signal circuitry [26,72,76,77] (Figure 1C). Interestingly, SKP2 molecules are sustained by TRβ2 that antagonizes the tumor-suppressive function of TRβ1 to promote development of *RB1*-deficient malignancies [71,78].

TRβ2 and TRβ1 are encoded by the same TRβ gene but use different promoters, resulting in distinct N-terminal coding sequences [71]. TRβ1 suppresses SKP2 to prevent S-phase entry and progression and thus acts as a safeguard against tumorigenesis associated with *RB1* loss [71]. In contrast, a new oncogenic isoform of TRβ2, TRβ2-46, increases expression and stability of the SKP2 protein, which counteracts the TRβ1 tumor-suppressive function and thus promotes the proliferation of retinoblastoma cells [68] (Figure 1C). Counteraction between TRβ1 and TRβ2 also confers minimal genomic instability, which may be responsible for decreased aneuploidy, so that tumors escape from possible death due to chromosomal abnormalities in rapidly proliferating retinoblastoma cells [25].

MDM2 is intrinsically expressed at high levels in maturing human cone and retinoblastoma cells, which is regulated by cone transcription factor RXRγ [46,69] (Figure 1C). MDM2 complexing with MDM4 may facilitate p53 degradation in primary retinoblastoma where *TP53* mutation is very rare [79], contributing to resistance against cell death [42] (Figure 1C). Additionally, an MDM2 inhibitor, p14^ARF^ (CDKN2A^ARF^), is repressed by miR24, thereby compromising the p53 response during retinoblastoma genesis [67,70] (Figure 1C). High levels of MDM2 in retinoblastoma cells suppress p14^ARF^-induced apoptosis [46]. MDM2 itself enhances mRNA expression and the translation of the MYCN oncoprotein that is responsible for p53-independent proliferation [69] (Figure 1C). MYCN is active in the developing retina to promote proper cell growth and proliferation, and downregulation is required to maintain cellular homeostasis in the adult retina [80]. MYCN is enriched in human retinoblastoma cells to promote tumor outgrowth [81,82,83], although how MDM2 regulates MYCN is unclear.

If cone cells are the cell-of-origin, pRB depletion and cone-specific signal circuitry may enable proliferation in retinoblastoma, then how could an arrest in retinoma, which is thought to be a precursor of retinoblastoma [3], be explained? (Figure 1B). Retinoma is benign and can coexist with retinoblastoma [3,51]. Retinoma cells produce senescence proteins and are incapable of proliferating, thereby blocking malignant transformation [3,17,51]. A stable form of retinoma is uncommonly observed in clinics [3]. As mentioned above, an increase in genomic instability is proposed as an oncogenic driver [33,45]. Progressive genetic and epigenetic alterations superimposed on the cone circuitry may ultimately induce development of premalignant cells into highly proliferative retinoblastoma cells [3,5,17,46,51].

## 3. Disease Modeling

Retinoblastoma models are important to advance our understating of the tumor initiation and progression as well as assess therapeutic effects. Next, we review retinoblastoma models in terms of their derivation, characteristics, and advantages and limitations for studying the disease as well as therapeutic screening and testing.

### 3.1. Genetically Engineered Mouse Models (GEMMs)

Unlike the penetration of the germline *RB1* mutation in human retinoblastoma, mice cannot develop retinoblastoma with similar disruption of the *Rb1* gene, even if pRb is completely inactivated, because pRb-related proteins p107 (RBL1) and p130 (RBL2) compensate for functional loss of pRb [84,85]. Inactivation of either is required in addition to pRb loss for tumorigenesis [85,86] (Table 3). Combined losses of pRb and p130 robustly enhance tumor development with an increased number of mice carrying bilateral tumors and fast tumor formation compared with concomitant loss of pRB and p107 [50,85,86,87,88] (Table 3). This suggests that p130 is a stronger tumor suppressor. Additionally, tumors from pRb/p130 double-knockout mice exhibit less frequency of genomic alterations because of faster tumor development [89], which is consistent with less genomic instability of tumors diagnosed in very young patients. Tumor formation is dramatically accelerated and robust in triple-knockout mice, in which pRb and p107 are inactivated along with loss of either p130, p53, or Pten, and pRb/p107 double-knockout mice with conditionally overexpressed MDM4 (Table 3). This suggests that loss of p53 or Pten surveillance pathways and gaining oncogenic MDM4 expression enhance tumor formation but are not required for tumorigenesis.

A mouse model of *MYCN*-amplified retinoblastoma without an *RB1* mutation is generated by conditionally overexpressed MYCN in retinal cells with wildtype *Rb1*, but the tumors do not develop [83]. This conflicts with human tumorigenesis where high *MYCN* amplification is proposed as a driver in a rare form of retinoblastoma without *RB1* mutation [39]. In fact, retinoblastoma forms when the effect of overexpressed MYCN cooperates with pRb loss in mouse retinal cells. MYCN bypasses any need to inactivate p107 or p130 for tumor development [83] (Table 3).

Conditional knockout of *Rb1* occurs in retinal progenitor cells, according to a Cre driver in a Cre/Lox system for generating GEMMs [50,85,87,88,90] (Table 3). The affected cell types, either progenitors themselves or their differentiating progenies, may be the origin of tumors. Interestingly, the characteristics of horizontal and amacrine interneurons expressing syntaxin are common in retinoblastoma of GEMMs with distinct genotypes [50,85,86,87,90]. This supports the ability to proliferate and form metastatic lesions of horizontal interneurons deficient for pRb, p130, and p107 [87]. In contrast, pRb-depleted photoreceptors do not form retinoblastoma in GEMMs, even when photoreceptor cells are deficient for pRb, p107, and p53 [91]. Similarly, pRb-depleted mouse cone cells do not develop tumors, even when *MDM2* and *Mycn* are ectopically expressed in the cells to imitate human tumorigenesis [17]. This indicates that different susceptibilities to tumorigenesis may exist in mice and humans. The expression of human cone signal circuitry in mouse horizontal cells suggests that horizontal cells represent human cone cell counterparts and are the tumor cell-of-origin in mice [46]. Analyses of the epigenetic landscape further suggest that human tumors differ significantly from mouse retinoblastoma in which some candidate pathways for molecular targeted therapies of human retinoblastoma are not deregulated [92]. This becomes an important issue when candidate drugs showing therapeutic effects in preclinical models are not effective in humans.

### 3.2. Xenografts and Organoids

Xenograft models are generated through orthotopic or subcutaneous implantation of patient-derived tumor cells into immunodeficient animals. Subcutaneous xenografts are maintained by serial passaging from one mouse to another [94,95]. Orthotopic implantation, in which tumor cells are injected directly into the anterior or vitreous cavity, has a high success rate of model establishment compared with subcutaneous xenografting [94,95,96,97]. Additionally, orthotopic models have demonstrated tumor invasion into the retina, subretinal space, ciliary body, choroid, and sclera as well as dissemination into the brain [97]. Histologically, orthotopic xenografts replicate typical rosettes or undifferentiated features found in human retinoblastoma, whereas subcutaneous xenografts produce an undifferentiated phenotype, even though the original tumor tissues are classified as differentiated tumors [94,95,97]. This implies that the microenvironment in the eyes is important to successfully recapitulate the histological features of retinoblastoma.

Tumor organoids can be directly generated from chemotherapy-naïve retinoblastoma from enucleation [48]. The organoid models recapitulate the tumor tissues in terms of histological features, DNA copy number changes, as well as gene and protein expression [48]. Cone signal circuitry and the glial tumor microenvironment remain in organoids, and the drug responses of organoids mimic those of tumor cells in advanced disease [48]. Additionally, human retinal organoids generated from induced pluripotent stem cells (iPSCs), or embryonic stem cells (ESCs), are alternatives to the fetal retina which has limited availability [98,99,100,101,102]. The retinal organoids made deficient for *RB1* may provide a model to examine the initiation and development of tumors, such as the cancer cell-of-origin [17,26].

Somatic cells from patients with germline *RB1* mutations have been used to generate iPSC lines with heterozygous *RB1* deletion [103,104,105]. However, neither iPSCs with subsequent inactivation of the other *RB1* allele nor retinal organoids derived from *RB1*-deficient iPSCs have been reported. It would be interesting to determine whether retinal organoids can be generated from iPSCs with biallelic loss of *RB1* to examine malignant transformation. Nevertheless, homozygous knockout of *RB1* has been conducted in ESCs to model and study the genesis of trilateral retinoblastoma via teratoma formation [106,107]. However, it is unknown whether orthotopic transplantation and retinal differentiation prior to grafting are required to recapitulate retinoblastoma formation of *RB1*-deficient ESCs/iPSCs.

### 3.3. Advantages and Limitations

GEMMs spontaneously develop retinoblastoma and allow the study of early or advanced tumors, or both, as well as disease progression according to their genotypes (Table 3). However, human retinoblastoma genesis, including tumor cell-of-origin, is not accurately recapitulated in GEMMs, which might give irrelevant results of therapeutic testing. Xenograft models represent the advanced disease stage and better recapitulate human tumors; this is because the surgical samples are obtained from patients with advanced tumors and contain genetic lesions that occur in the disease. However, an appropriate mouse strain for setting up an orthotopic retinoblastoma model must be used to achieve the best engraftment rate and tumor growth [96]. Retinoblastoma organoids serve as a model for advanced tumors. However, the tumor microenvironment is incompletely present in the retinoblastoma organoids [48]. GEMMs are more suitable to examine the role of microenvironment, such as immune cells, on tumorigenesis and therapies. Because of the expandable source, tumor organoids are applicable to high throughput drug screening and testing, whereas GEMMs and orthotopic xenograft models are better for validating the therapeutic effects of drug candidates and testing methods of drug delivery. The combination of organoid and in vivo models has clear advantages for preclinical studies of therapeutics and treatments.

## 4. Treatments

Retinoblastoma treatment primarily aims to save the patient’s life and then their globe and vision. Although retinoblastoma is the most curable cancer with a high survival rate, advanced retinoblastoma limits globe and vision salvaging and risks the patient’s life because of metastasis. Next, we summarize the classification and staging systems and high-risk histopathological features of retinoblastoma, which are crucial for disease management and prognosis. Additionally, we discuss the current treatments, correlations between pathological risks and clinical features, which assist in guiding treatment and may facilitate globe preservation, and development of targeted therapeutics based on the biological mechanisms underlying the disease.

### 4.1. Classification and Staging Systems

The classification schemes have been developed to predict success of globe survival in intraocular retinoblastoma based on treatment modalities. In the external beam radiotherapy (EBRT) era, Reese and Ellsworth classification was widely used [108]. In the systemic chemotherapy era, the International Intraocular Retinoblastoma Classification (IIRC) was developed by Murphree [109] and modified into a scheme known as the Intraocular Classification of Retinoblastoma (ICRB) which is found to successfully predict the outcome of intravenous chemotherapy (IVC) [110]. These systems classify tumors, according to their size, location, and additional features including tumor seeds (refer to Fabian et al. 2018 [111] for further details), into groups A–E from very low to very high risk. However, significant discrepancies remain for group assignments using IIRC and ICRB, especially group D and E eyes, leading to inconsistency in clinical research reports and making comparisons difficult between treatment centers [112].

The International Retinoblastoma Staging System (IRSS) and Tumor, Node, Metastasis (TNM) staging system have been used to cover the whole spectrum of retinoblastoma. Recently, the American Joint Committee on Cancer (AJCC) developed the 8th version of AJCC retinoblastoma staging. This new version has acknowledged the importance of detecting the heritable trait (H) in addition to TNM and is proposed to predict globe salvage, metastasis and patient survival more accurately [113].

### 4.2. High-Risk Histopathological Features

Risk of tumor dissemination increases as growing tumors invade into the optic nerve and highly vascularized choroidal tissue. Tumor cells may spread into the cranial cavity, bone, bone marrow, or lymph nodes. An important histological feature examined in enucleated globes and considered as a high-risk factor for distal metastasis is retrolaminar optic nerve invasion including the tumor at the surgical margin and massive choroidal invasion at ≥3 mm in diameter [51,52,114,115,116]. Tumor invasion into the anterior segment and any degree of concomitant non-massive choroidal and prelaminar/laminar optic nerve invasions are considered as prognostic risk factors [51,52,114,115,116]. These high-risk histopathological features are indications for adjuvant chemotherapy at most clinics.

### 4.3. Current Treatments

Treatment of retinoblastoma is challenging and has been markedly developed in recent decades. In the 1960s, EBRT was extensively used for the treatment of intraocular retinoblastoma. However, with the advent of systemic chemotherapy, the popularity of EBRT has declined because of its side effects such as development of secondary malignancies and radiation-induced complications [117,118]. Systemic chemotherapy combined with adjunctive local treatment (laser photocoagulation, transpupillary thermotherapy, cryotherapy, and brachytherapy) has been widely used for the treatment of retinoblastoma. The most common regimen for systemic chemotherapy is vincristine, carboplatin, and etoposide (VEC regimen). With chemoreduction, the globe survival rates for IIRC group A, B, and C eyes were more than 90% [110]. The globe survival rate was 47% [110] for bilateral group D eyes treated with systemic chemotherapy alone and increased to 68% in combination with low-dose intensity-modulated radiation therapy [119]. Group E eyes are often considered unsalvageable because of the metastatic risk [110], although multimodality treatment lead to less enucleation [9].

Yamane et al., 2004, introduced the ophthalmic artery infusion technique to minimize systemic toxicity of chemotherapeutic agents and to obtain better tumor responses with the goal of eye retention [120]. The technique was developed further and is now known as direct intra-arterial chemotherapy (IAC) for treating advanced intraocular retinoblastoma [121]. A commonly used drug in IAC is melphalan with or without the combination of topotecan and/or carboplatin [18]. The ocular survival rate has improved significantly for IAC with 85% of group D eyes conserved [122]. However, IAC has a learning curve and requires a skillful neurointerventionist. These factors may restrict implementation of IAC in developing countries, while success in controlling tumors varies among retinoblastoma clinical centers using the same technique [18,123].

Vitreous seeds represent the main challenge in the management of retinoblastoma because avascular sites in the vitreous cavity can cause poor penetration of systemic chemotherapy. Even with IAC, approximately 30% of eyes are removed because of the presence of vitreous seeds [124,125]. Intravitreal chemotherapy injection (IViC) infuses a high concentration of chemotherapy into the vitreous cavity, but with a risk of extraocular tumor dissemination. Historically, IViC has been mainly reported for treating severe and monocular cases with recurrent retinoblastoma and extensive vitreous seeding [126]. Kaneko and Suzuki 2003 pioneered injecting melphalan intravitreally to control retinoblastoma with vitreous seeding, in combination with whole eye hyperthermia, and reported a globe survival rate of 51.3% [127]. However, there are reports on retinal toxicity following intravitreal melphalan injection in patients or in a preclinical rabbit model, shown by reduction in electroretinogram (ERG) components [12,13]. Munier et al., 2012, later demonstrated the safety profile of intravitreal injections for retinoblastoma using an anti-reflux procedure and sterilization of the needle tract [128]. Additionally, examining RNA reflux of retinoblastoma can be used to assess potential extraocular dissemination after IViC [129]. Melphalan (20–30 µg) is commonly used for IViC [18]. Topotecan is used in combination with melphalan for refractory vitreous seeds [12,130,131] or as monotherapy [132]. This technique has been widely adopted and proven safe [12,133] and effective with 69–100% rates in controlling vitreous seeds [130,131,134,135,136].

While there has been increased success in salvage of globes, there remains significant debate regarding whether the salvage of these advanced eyes places the child at risk for metastatic disease given that there may be choroidal or optic nerve invasion that cannot be identified clinically. A better means of identifying which eyes are safe to salvage and which are not is still a critical area of need in the treatment of retinoblastoma. A systematic review of IAC in 613 patients reported that at least 20 children had metastases and 14 died [137]. However, this may not solely reflect the results of IAC because the studies included bilateral retinoblastoma cases that also received radiation therapy and had a second malignant neoplasm [137]. However, a retrospective single center comparative review of 48 sporadic unilateral retinoblastoma group D patients treated primarily with IVC and IAC, showed no extraocular disease, metastases, or long-term systemic complications in both groups at a mean follow-up of 105 months [138].

The overall survival of enucleation alone in unilateral, non-heritable cases without extraocular extension is 85–90% [139,140]. Enucleated eyes need meticulous evaluation for high-risk pathological features by a histopathologist trained in the evaluation of retinoblastoma globes. Untreated patients with high-risk histopathological features develop systemic metastases in 24% of cases [141]. Post-enucleation adjuvant chemotherapy has been recommended for patients with retinoblastoma manifesting high-risk features to prevent systemic metastases that can cause death [141]. Kaliki et al. 2011 analyzed 512 ICRB group E eyes that underwent enucleation and received post-enucleation adjuvant chemotherapy and found no evidence of systemic metastasis at a mean follow-up of 66 months [142]. Furthermore, a multicenter study in Latin America included 175 retinoblastoma patients and classified enucleated patients as high- and low-risk groups with post-enucleation systemic chemotherapy administered only in the high-risk group [10]. Overall survival was 100% in the low-risk group without receiving systemic chemotherapy and 95% in the high-risk group. The authors suggested that adjuvant chemotherapy may not be necessary for low-risk patients but benefits patients with high-risk retinoblastoma in preventing extraocular relapse [10].

The role of systemic chemotherapy in prevention of pineoblastoma or second primary neoplasm in germline retinoblastoma patients is still debatable, while deaths related to toxicity in chemotherapy remain a major concern [18]. Thus, there is debate about the practice preferences among leading institutions for retinoblastoma treatment regarding genetic mutation, laterality, the preferred treatment modalities for advanced retinoblastoma, and the use of postenucleation adjuvant chemotherapy [143].

Retinoblastoma is a rare cancer, and therefore it is difficult to perform prospective randomized control trials to compare the various outcomes of each treatment modality. The decision for management of each patient should be individualized based on interpretation of the available published data, available treatment modalities in each clinic, and the opinion of the patient’s family on treatments.

### 4.4. Clinicopathological Correlations

Group D and E eyes are associated with high-risk features, which warrant adjuvant chemotherapy [52,114,115,144,145,146]. However, not all enucleated globes show high-risk features, and such patients may not need adjuvant chemotherapy [11,51,52,114,115,144,145,146,147] (Table 4). This is particularly true for group D eyes with only 10–17% of globes carrying high-risk pathology [114,115,144,146,148,149]. It is therefore feasible, if needed, to save the affected eyes such as in patients with bilateral retinoblastoma [150]. However, globe-preserving methods of treatment must not risk tumor spread compared with primary enucleation. Studies have demonstrated that globes can be saved without an increased risk of metastatic disease or orbital recurrence by systemic chemoreduction and subsequent enucleation for poor responses [8,145]. Globe salvage can be achieved for advanced group D and E eyes with a lower rate for group E eyes, while some studies report vision salvage [9,134,138,151]. Clinical indications are needed to predict whether these high-risk pathological features present without histopathological examination, so that the globe can be spared or treated promptly.

Clinicopathological correlations have been used to define clinical features that can predict high/low-risk histopathological features (Table 4). However, it is important to note that, compared with secondarily enucleated eyes, primarily enucleated eyes generally harboring high-risk histopathological features [8,152] are only included in correlation studies because chemotherapy may downgrade the risk features [152]. Using this criterion, many studies have shown that group E eyes correlate significantly with high-risk pathology (Table 4). Due to the risk of metastatic spread in high-risk eyes, any attempts at salvaging group E eyes should be done with extreme caution; at most centers it is only undertaken in the setting of bilaterally advanced disease and even then, with the caveat that enucleation will be performed for any lack of therapeutic response. Additionally, high-risk pathology appears to correlate with the following clinical features: raised intraocular pressure, secondary glaucoma, delayed time to treatment, iris neovascularization, a shallow anterior chamber, or the absence of vitreous seeds in group D eyes (Table 4). Minimally disseminated disease, which is indicated by retinoblastoma-associated mRNA detected in bone marrow or cerebrospinal fluid, is likely to occur in patients with glaucoma and Group E eyes [153,154]. In contrast, macular sparing, optic nerve visibility, or retinal detachment in less than one quadrant represent low-risk histopathology in advanced tumors that have a high likelihood of successful eye salvage with very low risk of metastatic disease [149]. However, globes that fail preservation by any means must be promptly enucleated and further treated appropriately; this is because any delay can result in poor outcomes [148,152].

### 4.5. Future Treatments

Cone-associated molecules TRβ1 and TRβ2 are linked with pRb pathway at least through controlling SKP2 activity in the pRb-SKP2-p27 axis (Figure 1C), while some other molecules, such as MDM2, E2F1, and SYK, also promote tumorigenesis. This knowledge would facilitate designing targeted approaches that are expected to be effective and less toxic.

SKP2 activity is enriched in many malignancies [155]. Functional loss of SKP2 has a synthetic lethal effect in *RB1*-deficient tumors including retinoblastoma [71,76,77,156]. SKP2 inhibitors that prevent SKP2 binding to the SCF (Skp1-Cullin1-F-box) core subunit inhibit proliferation of retinoblastoma cells, but the potency of currently synthesized inhibitors is poor [157]. Pevonedistat (MLN4924) suppresses NEDD8-activating enzyme E1 activity in neddylation of Cullin1, thereby dampening SCF activity [158]. Pevonedistat tested in clinical trials for treatment of many cancers potently suppresses the growth of retinoblastoma with minimal off-target effects via intravitreal delivery in orthotopic xenograft models [157,158]. The drug exerts a broad range of activities from cytostatic to lethal in multiple retinoblastoma cell lines, regardless of the *RB1* or *MYCN* status [157]. Thus, inactivation of SCF^SKP2^ complexes provides a concept of using synthetic lethality as a therapeutic strategy against retinoblastoma. However, no chemically synthesized probes have been qualified as validated pharmaceutical hit compounds to suppress SKP2 activity [159].

Increased expression of MDM2 or MDM4 can result in inactivation of the p53 pathway. A small molecule inhibitor of MDM2–p53 interaction, nutlin-3, induces p53-mediated death of retinoblastoma cells harboring wildtype p53 [42]. Nutlin-3 disrupts both MDM2-p53 and MDM4-p53 interactions in retinoblastomas with less efficiency in the latter. Moreover, a synthesized antagonist, SJ172550, of the MDM4-p53 interaction effectively kills retinoblastoma cells with elevated MDM4 and has additive effects with nutlin-3 [160]. However, an independent study has suggested that MDM2 promotes outgrowth of retinoblastoma through promoting MYCN expression and should be primarily targeted [69]. These findings suggest that MDM2-targeting therapeutics assist in retaining p53 tumor-suppressive activity while inhibiting MYCN to treat retinal tumors.

Histone deacetylases (HDAC) inhibitors currently approved by the Food and Drug Administration (FDA) or in clinical trials for treatment of multiple cancers [161] have been found to selectively kill cells with elevated E2F1 by induction of pro-apoptotic BH3-only protein Bim [162]. HDAC inhibitors suppress the growth of retinoblastoma in which *RB1* inactivation leads to increased E2F activity in transgenic and xenograft models with minimal side effects on ocular tissues [163]. Additionally, BIRO1, a synthesized peptide emulating the effect of the BH3 domain, induces cell death in retinoblastoma cells while sparing a photoreceptor cell line [164]. These inhibitors may represent good candidates to selectively eradicate tumor cells. Additionally, oncolytic adenovirus designed to selectively replicate in and lyse tumor cells with high expression of free E2F1 exhibits anti-tumor activity in human retinoblastoma models and phase I clinical trials [165]. This approach appears to be a new candidate therapy for eyes afflicted by chemoresistant retinoblastoma [165].

Epigenetically upregulated SYK expression is evident in retinoblastoma and required for tumor cell survival [5]. SYK silencing increases apoptosis of tumor cells, while in xenograft models, SYK inhibitors induce cell death of retinoblastoma through decreased MCL1, the only anti-apoptotic member of the BCL2 family up-regulated in retinoblastoma [5]. Interestingly, SYK is not expressed in the normal retina. Thus, SYK is a promising new target for selectively killing tumors cells while sparing retinal tissue.

Gene expression levels of these target molecules may be different in individual tumors and need to be determined from tumor biopsies to enable personalized therapy. However, understanding risk profiles of tumor biopsies is essential before the findings can be translated clinically. Given the prohibition to biopsy in this cancer there has been progress using both blood and the aqueous humor, however none are used clinically at this time [166,167,168,169].

## 5. Conclusions and Future Perspectives

Retinoblastoma is the most curable cancer with a high survival rate if diagnosed and treated early. Currently, much emphasis has been placed on preserving eyes that previously were enucleated because of advances in knowledge and technology first with systemic chemotherapy and then local drug delivery including intra-arterial or intravitreal chemotherapy. The latter has shown great success for globe retention, even in the advanced groups. Great efforts have been made to detect retinoblastoma at risk of metastasis using histopathological and clinical features or mRNA. However, predictive biomarkers that are more sensitive and specific are desired to identify metastasis preoperatively, so that high-risk children may be more readily identified and treated accordingly, while a conservative approach would be advisable to patients without dissemination. Moreover, the toxicity of chemotherapy regardless of delivery routes indicates the need to develop novel targeted drugs, which will require more basic and translational research. It appears that human cone cells are sensitized to malignant transformation, in which cone signal circuitry is oncogenically activated in response to *RB1* inactivation. Cone signal circuitry and its connecting molecules with oncogenic roles (Figure 1) represent a target for treatment of retinoblastoma. Disease progression appears to correlate with gaining genomic instability while losing cone signature and adoption of a less differentiated grade and anaplasia.

Evidence shedding light on tumor initiation, including the cell-of-origin, has become more available, but molecularly targeted therapy has not been developed for retinoblastoma. This may be attributed to too few patients to conduct clinical trials or interest from the pharmaceutical industry. Drug reprofiling provides an opportunity for the discovery of targeted therapeutics for such a rare disease. The advent of retinoblastoma organoids enables drug discovery in pathologically relevant and high-throughput assays. Molecularly targeted drugs should be more effective and less toxic, and the effects of drugs would be potentiated by local delivery. This may not only save lives, but may also spare eye(s), some sight, and the quality of life of retinoblastoma survivors.

## Figures and Tables

**Table 1:** Candidate drivers defined in a minimal region using arrays.

Reference	1q Gain	2p Gain	6p Gain	16q Loss	Eye No.	Array
Kooi et al. 2016 [33]	*CRB1*, *NEK7*, *MIR181*	*MYCN*	*SOX4*, *DEK*	*RBL2*	45	SNP
Mol et al. 2014 [31]	*KIF14*, *MDM4*, *LRNN2*, *ZNF281*	*MYCN*, *DDX1*	*DEK*, *E2F3*, *TNF*, *KIF13A*, *TDP2*, *CAP2*, *NUP153*, *SOX4*, *ID4*	*CDH11*, *CDH13*, *RBL2*, *MBTPS1*, *ZCCHC14*, *ZDHHC7*	21	SNP
Sampieri et al. 2009 [4]	*MCL1*, *SHC1*, *MUC1*	*MYCN*, *DDX1*	*IRF4*, *DEK*, *PIM1*, *E2F3*, *CCND3*	*CYLD*, *RBL2*	18	CGH
Zielinski et al. 2005 [32]	*SHC1*, *MDM4*, *GAC1*	*MYCN*	*TNF-alpha*, *HLA gene cluster*	*RBL2*	17	CGH
Chen et al. 2001 [27]	*LRNN2*, *REN*, *GAC1*	*MYCN*	*E2F3*, *ID4*	*CALB1*, *CBFB*, *CDH1*, *CDH11*, *CDH13*, *CDH15*, *CDH16*, *CDH3*, *CDH5*, *CDH8*, *E2F4*, *MAF*, *ZFHX3*	50	CGH
Herzog et al. 2001 [28]		*MYCN*		*RBL2*	26	CGH

**Table 2:** Associations between genomic and transcriptomic features and clinicopathological characteristics.

Clinicopathological Characteristics	Genomic Instability	Photoreceptor Gene Signature	
		High	Low
Laterality [33,45]	Unilateral	Bilateral	Unilateral
Tumor grade * [33,44,45]	Less differentiation	Differentiation	Less differentiation
Age at diagnosis/enucleation [33,45,51,52]	Older	Younger	Older
High-risk features (optic nerve/choroid invasion) [33,44,45]	Not associated	Not associated	Not associated
Tumor volume [33,45]	Not associated	Smaller	Larger
Tumor location [45,53]	N/a	Central retina	Peripheral retina/entire retina
Eye size [53]	N/a	Smaller	Larger
Number of lesions [33,53]	Not associated	1–5 lesions	>5 lesions
Chemotherapy sensitivity [45]	N/a	Less sensitivity	More sensitivity

N/a: not available; * Presence/absence of Flexner–Wintersteiner rosettes.

**Table 3:** Genetically engineered mouse models of retinoblastoma (RB).

Genotype of the Genetically Engineered Mouse Model	Characteristics	Study Objective
*Rb1/p107* DKO [50,88,90]	68% of mice with RB develop by 280 days and 22% of mice carry bilateral RB	Additional genes required in cooperation with pRb loss for tumorigenesis and early RB
*Rb1*/*p130* DKO [50,85,88]	85% of mice with RB develop by 128 days and 28% of mice carry bilateral RB	Early and advanced RB
*Rb1*/*p107*/*p130* TKO [50]	100% of mice with RB develop by 80 days and 83% mice of carry bilateral RB	Very aggressive RB
*Rb1*/*p107*/*p53* TKO [50,90]	98–100% of mice with RB develop by 100 days and 65% of mice carry bilateral RB	Advanced and aggressive RB
*Rb1*/*p107* DKO/*MDMX* Tg [50]	90% of mice with RB and 63% of mice carry bilateral RB	Advanced and aggressive RB
*Rb1*/*p107*/*Pten* TKO [93]	100% of mice with bilateral RB develop by 30 days	Tumor progression related to the PI3K/AKT pathway
*Rb1* KO/*MYCN* [83]	100% of mice with RB develop by 54 days	Oncogenic effects of MYCN on RB

**Table 4:** Clinicopathological correlations of retinoblastoma.

Histopathological Features at Primary Enucleation (% Enucleated Eyes with Risk Features)	Total Eye No.	Correlated Clinical Features (% Patients with Described Features Exhibiting Clinicopathological Correlations)
LR (89.5%) * [149]	38	Macular spare (26%) Optic nerve visibility (42%) <1 quadrant of retinal detachment (22%) (studied in group D eyes)
HR (47%) [145]	96	Raised intraocular pressure (>34 mmHg) in group E eyes (100%)
HR (12.5%) [144]	40	No vitreous seeds in group D eyes (20%)
HR (36%) [115]	403	Group E eyes (39%) Delayed time to treatment >6 months (63%) Secondary glaucoma (64%)
HR (23%) [114]	519	Group D (17%) and E (24%) eyes
HR (64%) [52]	76	Iris neovascularization (63%) Raised intraocular pressure (>21 mmHg) (63%) ** Shallow anterior chamber (26%) (studied in group E eyes)
HR (25%) [146]	67	Group E eyes (50%)

Low-risk histopathological feature (LR) is defined by intraocular tumor(s) with local invasion or intraocular tumor(s) without local invasion, focal choroidal invasion, or prelaminar or intralaminar involvement of the optic nerve head, while high-risk histopathological feature (HR) is defined by presentation of one or more of the HR features in enucleated eyes (see HR in 4.2), * No correlation between HR and clinical features, ** Not consistent with Wilson et al., 2011 [146].
